# 34.4 IMPROVING THE DETECTION OF INDIVIDUALS AT RISK OF PSYCHOSIS IN THE COMMUNITY: A NEURODEVELOPMENTAL PERSPECTIVE

**DOI:** 10.1093/schbul/sby014.145

**Published:** 2018-04-01

**Authors:** Monica Calkins, Tyler Moore, Daniel Wolf, Theodore Satterthwaite, David Roalf, Bruce Turetsky, Christian Kohler, Kosha Ruparel, Ruben Gur, Raquel Gur

**Affiliations:** University of Pennsylvania

## Abstract

**Background:**

Increasing our ability to identify youths at risk of psychosis in the general public is a key step towards an improved ability to prevent the disorder. Prospective evaluation of youths with early psychotic-like experiences can enrich our knowledge of clinical, biobehavioral and environmental risk and protective factors associated with the development of psychotic disorders.

**Methods:**

By using a neurodevelopment prospective cohort study we aimed to investigate the predictors of psychosis spectrum features among US youth. This is the first large systematic study to evaluate subclinical symptoms in the community. From a Time 1 screen of 9,498 youth (age 8–21) from the Philadelphia Neurodevelopmental Cohort, a subsample of participants was enrolled based on presence or absence of psychosis spectrum symptoms to participate in an approximately 2-year (n=503, mean age=17) and/or 4-year (n=313; mean age=19) follow-up assessment. Participants were administered the Structured Interview for Prodromal Syndromes, conducted blind to initial screen status, along with the Schizotypal Personality Questionnaire and other clinical measures, computerized neurocognitive testing, and neuroimaging. Age normative references scores of baseline psychosis screening measures were applied to inform interpretation of psychosis symptom endorsements. Clinical and demographic predictors of symptom persistence were examined using logistic regression.

**Results:**

At 4-year follow-up, psychosis spectrum features persisted or worsened in 58% of youths endorsing symptoms at baseline. Among youths assessed at all three time-points (n=197), 54% showed temporal stability in presence or absence of psychosis spectrum symptoms, while the remainder exhibited varying patterns of symptom emergence, remission and re-occurrence over time. Baseline depression and social/occupational dysfunction were significant predictors of the occurrence of psychosis spectrum symptoms at either follow-up. Preliminary data on neurocognition, and brain structure and function, will be also discussed with the ultimate aim of integrating them with clinical data, to provide early indices of symptom persistence and worsening in youths at risk for psychosis.

**Discussion:**

Together, our findings indicate that varying trajectories of psychosis spectrum symptoms are evident early in US youth representative of the general community, supporting the importance of investigating psychosis risk as a dynamic developmental process.

